# Hybrid oxide brain-inspired neuromorphic devices for hardware implementation of artificial intelligence

**DOI:** 10.1080/14686996.2021.1911277

**Published:** 2021-05-14

**Authors:** Jingrui Wang, Xia Zhuge, Fei Zhuge

**Affiliations:** a School of Electronic and Information Engineering, Ningbo University of Technology, Ningbo, China; b Ningbo Institute of Materials Technology and Engineering, Chinese Academy of Sciences, Ningbo, China; c Center of Materials Science and Optoelectronics Engineering, University of Chinese Academy of Sciences, Beijing, China; d Center for Excellence in Brain Science and Intelligence Technology, Chinese Academy of Sciences, Shanghai, China

**Keywords:** Artificial intelligence, brain-inspired neuromorphic computing, memristor, artificial synapse, hybrid oxide, 40 Optical, magnetic and electronic device materials, 201 Electronics / Semiconductor / TCOs, 306 Thin film / Coatings

## Abstract

The state-of-the-art artificial intelligence technologies mainly rely on deep learning algorithms based on conventional computers with classical von Neumann computing architectures, where the memory and processing units are separated resulting in an enormous amount of energy and time consumed in the data transfer process. Inspired by the human brain acting like an ultra-highly efficient biological computer, neuromorphic computing is proposed as a technology for hardware implementation of artificial intelligence. Artificial synapses are the main component of a neuromorphic computing architecture. Memristors are considered to be a relatively ideal candidate for artificial synapse applications due to their high scalability and low power consumption. Oxides are most widely used in memristors due to the ease of fabrication and high compatibility with complementary metal-oxide-semiconductor processes. However, oxide memristors suffer from unsatisfactory stability and reliability. Oxide-based hybrid structures can effectively improve the device stability and reliability, therefore providing a promising prospect for the application of oxide memristors to neuromorphic computing. This work reviews the recent advances in the development of hybrid oxide memristive synapses. The discussion is organized according to the blending schemes as well as the working mechanisms of hybrid oxide memristors.

## Graphical abstract

**Figure:**
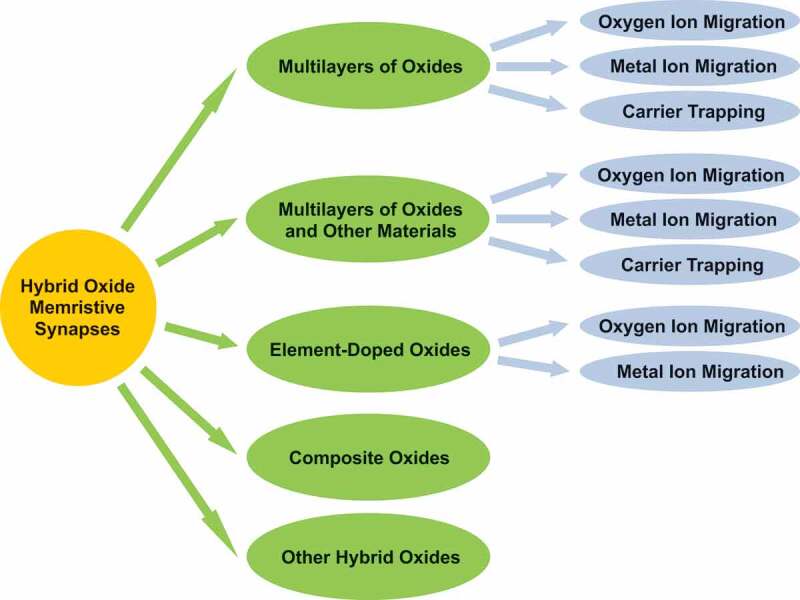

## 1. Introduction

Artificial intelligence is the ability of a machine to mimic the capabilities of the human mind, such as learning from past experience, recognizing objects and understanding language. Today, artificial intelligence is part of our everyday lives, including machine vision, real-time recommendations and virus/spam prevention. The state-of-the-art artificial intelligence technologies are mainly based on deep artificial neural networks running on conventional computers with classical von Neumann computing architectures, in which the memory and processing units are separated thus resulting in an enormous amount of energy and time consumed in the data transfer process. This is so-called von Neumann bottleneck. The human brain can be regarded as an ultra-highly efficient biological computer with a non-von Neumann architecture, which has approximately 10^11^ nerve cells linked together by 10^14^‒10^15^ synapses that brain signals travel through. Due to the highly-parallel and event-driven scheme of computation, the human brain is far superior to state-of-the-art computers in performing complicated learning and cognitive tasks [1].

Inspired by the human brain, neuromorphic computing, a technology for hardware implementation of artificial intelligence, is gaining more and more attention [2–7]. It mimics the neural structure and operation of the human brain at the physical level and therefore can perform advanced computation fast and efficiently. Artificial synapses are the main component of a neuromorphic computing system. The regulation of synaptic weight is the basis of computation. Artificial synapses have been developed by different schemes including complementary metal-oxide-semiconductor integrated circuits [8,9], transistors [10,11], memristors [12–19] and spintronic devices [20]. Among them, memristors are considered to be a relatively ideal candidate for artificial synapse applications due to their high scalability and low power consumption.

A memristor is generally a two-terminal electronic element with conductance or resistance that varies nonlinearly with the amount of charge flowing through it and can be remembered when the external electric power is turned off [21]. It demonstrates a ‘fingerprint’ featured by a pinched hysteresis loop in the first and the third quadrants of the current‒voltage (I–V) plane [22]. The memristor was predicted by Leon Chua in 1971 [23]. A research team from HP Labs correlated memristors to existing resistive switching devices in 2008 [24]. From this perspective, the first realization of the memristor can trace back to 1962 in which hysteretic resistive switching was observed in different oxide diodes [25]. Then, the resistive switching phenomena attracted much attention until the mid-1980s [26–29]. The current period of elevated activity of the corresponding research was triggered by Asamitsu et al. [30], Kozicki et al. [31] and Beck et al. in the late 1990s [32]. Generally, memristors consists of an insulating or semiconducting thin film sandwiched between two electrodes. The resistance of a memristor can be electrically tuned via various mechanisms, such as field-driven ion [33–39] or electron [40,41] behaviour or current-induced phase change [42,43], depending on the film and electrode materials used. Multi-level resistance states can serve as synaptic weights.

Till now, a large number of insulating or semiconducting materials have been employed as functional layers of memristive devices, such as oxides [24–27,30,32–37], chalcogenides [15,31,44], organic materials [45] and carbon [46,47]. Among these materials, oxides are most widely used due to the ease of fabrication and high compatibility with complementary metal-oxide-semiconductor processes. The resistive switching of oxide memristors generally originates from stochastic formation/rupture of nanoscale conducting filaments, which inevitably induces large device-to-device and cycle-to-cycle performance variations [6]. It is a severe obstacle to realizing large scale artificial neural networks. The utilization of oxide-based hybrid structures, e.g. oxide bilayers [48], can effectively improve the stability and reliability of memristors and therefore offers a promising prospect for the application of oxide memristors to neuromorphic computing. In this review, we discuss the recent advances in the development of hybrid oxide memristive synapses, mainly focusing on their blending schemes and working mechanisms.

## 2. Multilayers of oxides

### 2.1. Oxygen ion migration

**Figure 1.**
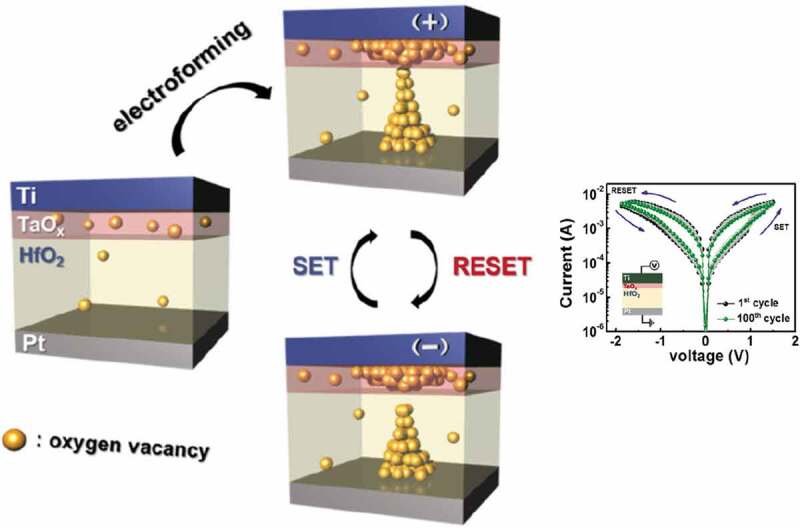
Schematic illustration of the resistive switching mechanism of HfO_x_/TaO_y_ memristive synapses. The inset shows the typical bipolar resistive switching behavior of a device. Reproduced with permission [48]. Copyright 2018, IOP Publishing Ltd

Generally, external electric stimuli will induce the drift of oxygen ions in oxide memristors, leading to the formation/rupture of nanoscale conducting filaments mainly composed of oxygen vacancies. Compared to single-layered oxide memristors, the devices based on a bilayer structure demonstrate more reliable performance likely due to a decrease in the stochasticity of the filament formation/rupture occurring at the interface between two oxide layers (Figure 1) [48]. Herein, electroforming (EF) refers to the switching process from the pristine high resistance state (OFF-state or HRS) to a low resistance state (ON-state or LRS). RESET and SET refer to the following ON-to-OFF and OFF-to-ON switching processes, respectively. A TaO_x_ insert layer can also act as the thermal enhanced layer to improve the uniformity of oxygen vacancy distribution [49]. The addition of a highly conductive Sn-doped In_2_O_3_ (ITO) layer to oxide memristors can enhance the linearity, symmetry and long-term stability of resistance modulation given that the ITO layer serves as a reservoir of oxygen ions drifted from the switching layer [50]. It has also been found that Ar plasma treatment of the surface of bilayered oxides can introduce additional oxygen vacancies into oxides, which are favorable for the formation of homogeneous and single conducting filament comprising oxygen vacancies [51]. Therefore, Ar plasma-treated devices show the improved uniformity of resistance change. Given the advantages of bilayered oxide memristors, a large number of bilayer structures have been developed including HfO_x_/TiO_y_ [52,53], HfO_x_/AlO_y_ [54–62], HfO_x_/BiFeO_3_ [63], HfO_x_/CeO_y_ [64], HfO_x_/TaO_y_ [48,49,55,65,66], HfO_x_/HfO_y_ [67], HfAlO_x_/TaO_y_ [68–70], TaO_x_/TaO_y_ [71–74], TaO_x_/AlO_y_ [75–79], TaO_x_/TiO_y_ [80–82], TaO_x_/ZnO [83], TaO_x_/InGaZnO_y_ [51], AlO_x_/AlO_y_ [84], AlO_x_/TiO_y_ [85–91], AlO_x_/ZnO [92], AlO_x_/NbO_y_ [93], TiO_x_/InGaZnO_y_ [94], ZnO/SiO_x_ [95], ZnO/MgO [96], InGaZnO_x_/InGaZnO_y_ [97,98], InGaZnO_x_/N-doped InGaZnO_y_ [99], ITO/CeO_2_ [50] and Sr_2_IrO_4_/BaTiO_3_ [100].

A trilayer structure of TiO_x_/HfO_y_/TiO_x_ has been proposed to reduce the device power. The resistive switching occurs in the HfO_x_ layer while the TiO_x_ layer acts as a resistor [101]. The trilayer configuration results in a low operation current and high ON/OFF ratio due to the existence of two TiO_x_ resistors. Such memristive devices present high symmetry and linearity of the resistance variation. A four-layer structure of HfO_x_/TiO_y_/HfO_x_/TiO_y_ has also been designed for memristive synapses [102].

Although a non-filamentary switching mechanism, i.e. uniform movement of oxygen ions at the electrode/oxide interface under external electric stimuli, has been suggested for some oxide memristors [103,104], the possibility of forming conducting filaments at the interface cannot be excluded given environmental perturbations, e.g. irregular surface morphology of electrodes which may result in non-uniformly distributed electric field. Therefore, it could be argued that the formation of nanoscale conducting filaments mainly composed of oxygen vacancies should be taken into account when investigating the resistive switching mechanism of oxide memristors with inert electrodes.

**Figure 2.**
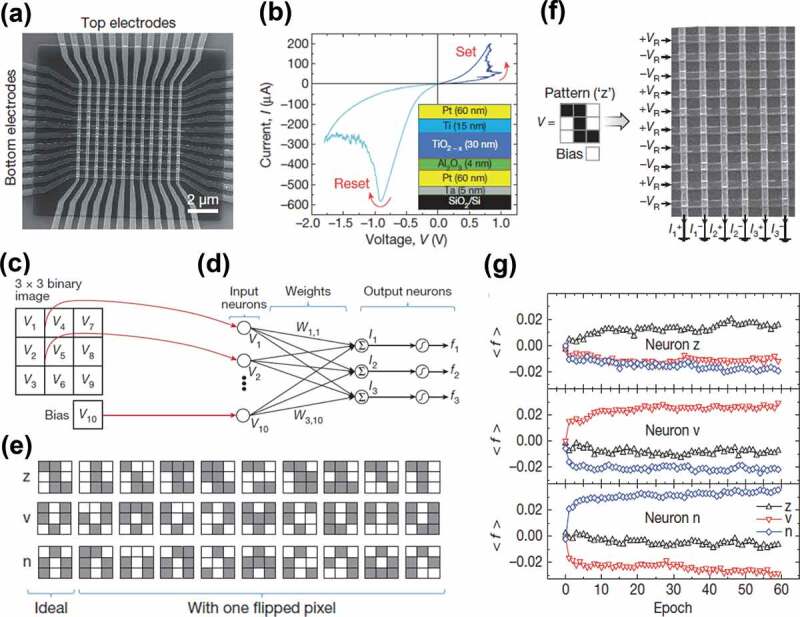
Crossbar array of TiO_x_/AlO_x_ memristive synapses and its pattern classification function. (a) 12 × 12 memristive crossbar. (b) Typical I–V curves of a device after the electroforming process. The inset schematically presents the device structure. (c) Input image where each pixel is represented by a voltage. (d) Schematic illustration of a single-layer perceptron for classification of 3 × 3 binary images. (e) The input pattern set used in the experiment. (f) Classification operation for the letter ‘z’. The black or white pixel is represented by an input signal of +*V*_R_ or – *V*_R_, respectively. (g) Output signal evolution. The classification is successful when the output signal (*f*) of the correct pattern class is larger than other outputs. Reproduced with permission [85]. Copyright 2015, Springer Nature

Memristor crossbar arrays based on bilayered oxides have been constructed and neuromorphic computing has been successfully performed. As an example, a 12 × 12 crossbar with TiO_x_/AlO_y_-based memristive synapses can be used to perform pattern classification (Figure 2(a**,b)**) [85]. The devices show highly nonlinear I–V curves and a high uniformity of the resistive switching parameters (Figure 2(b)). The memristor array is employed to construct a single-layer perceptron with ten inputs and three outputs, connected with thirty synaptic weights (Figure 2(c,d)). The perceptron’s output *f*_i_ (i = 1, 2, 3) are calculated as nonlinear activation functions *f*_i_ = tanh(*βI*_i_) of the vector-by-matrix product components *I*_i_ = *W*_i1_*V*_1_ + *W*_i2_*V*_2_ + *W*_i3_*V*_3_ + *W*_i4_*V*_4_ + *W*_i5_*V*_5_ + *W*_i6_*V*_6_ + *W*_i7_*V*_7_ + *W*_i8_*V*_8_ + *W*_i9_*V*_9_ + *W*_i10_*V*_10_, where *V*_1_‒*V*_9_ are the input signals, *V*_10_ is a constant bias, *β* is a parameter tuning the function’s nonlinearity, and *W*_i1_‒*W*_i10_ are tunable synaptic weights. The perceptron can be used to physically perform the classification of 3 × 3-pixel black-and-white images into three classes (stylized letters ‘z’, ‘v’ and ‘n’) with nine inputs corresponding to the pixel values. The network is tested on a set of thirty patterns consisting of those three stylized letters as well as three sets of nine noisy versions of each letter (Figure 2(e)). The black or white pixel is represented by an input signal of +0.1 or – 0.1 V, respectively. Each synapse comprises two memristors. Differential current output signal is achieved according to Ohm’s law. Figure 2(f) shows the classification operation for the letter ‘z’. The network is trained in situ based on the Manhattan update rule, a variation of the delta rule of supervised training. At each epoch, patterns from the training set are successively applied to the input and the corresponding outputs are used to calculate the delta-rule weight increments. Once all patterns of the training set have been applied and all delta-rule weight increments have been calculated, the synaptic weights are adjusted using the Manhattan update rule. When the memristors are initialized somewhere in the middle of their resistance range, the satisfactory classification is realized after about twenty training epochs (Figure 2(g)).

### 2.2. Metal ion migration

When the electrodes of oxide memristors are made of active metals such as Ag and Cu, redox reactions likely occur at the electrode/oxide interface upon electrical bias resulting in the formation of metal ions, e.g. Ag^+^ or Cu^2+^. The metal ions are drifted into the oxide layer under the electric field and reduced to metal atoms by harvesting electrons in the oxide or at the oxide/electrode interface, thus causing the growth of nanoscale metal filaments. The following resistive switching originates from the formation/rupture of metal filaments. Besides the commonly used Ag and Cu, some of the other metals, e.g. Ta [105], can also be employed as the active electrode of oxide memristors. A Sub-10 nm Ta channel has been directly observed within the oxide layer in a Ta/HfO_2_/Pt memristor [105], which is responsible for the resistive switching. Another case is that the oxide layer itself contains movable metal ions or metal clusters and the conducting filaments are made from these metal ions or clusters. In some metal oxide thin films such as TaO_x_, HfO_x_ and TiO_x_, the migration of metal ions occurs under an electric field [33], thus causing the formation of metal filaments which contribute to the switching. It is believed that only a single complete metal filament will be formed in the device because the growth of other filaments will be effectively suppressed after the oxide layer is electrically shorted [44]. The multilevel resistive switching of metal filamentary memristors likely originates from direct tunneling between the growing/rupturing filament and the electrode [106].

**Figure 3.**
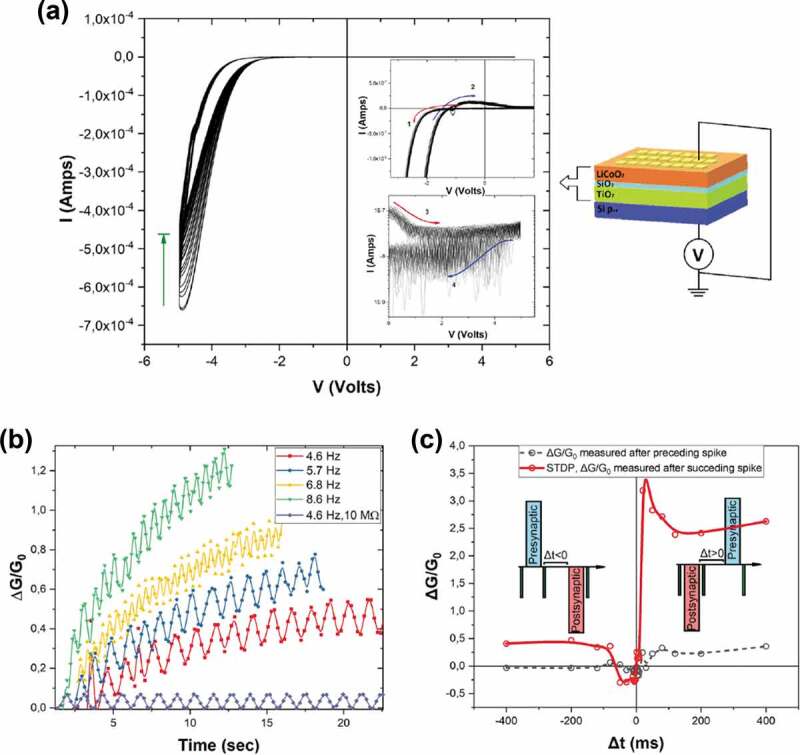
Synaptic functions emulated in LiCoO_2_/SiO_x_/TiO_2_ memristive devices. (a) Typical I–V characteristics of a device. The current hysteresis originates from the insulator-metal transition of LiCoO_2_. The non-zero crossing in the top inset can be attributed to the nanobattery effect. The bottom inset shows the RESET process during positive biasing. Schematic of the device structure is presented in the right inset. (b) Dependence of the conductance change on the stimulation frequency. (c) STDP bahavior. Reproduced under the terms of the CC-BY Creative Commons Attribution 4.0 International License [110]. Copyright 2020, The Authors, published by Springer Nature

A SiO_2_/Ta_2_O_5_ heterojunction has been designed to control the rupture of Ag filaments [107]. The growth direction of the filaments is controlled by the SiO_2_ layer and the filament rupture is controlled by the ultrathin Ta_2_O_5_ layer. After the initial electroforming process, the subsequent formation/rupture of Ag filaments is confined in the Ta_2_O_5_ layer, thus leading to low operation voltages and high stability and uniformity of resistive switching. It has also been found that the staking sequence of different oxide layers has significant influence on resistive switching characteristics of oxide memristors based on metal filaments. As an example, Ag/SnO_2_/InGaZnO_x_/Pt and Ag/InGaZnO_x_/SnO_2_/Pt devices demonstrate typical bipolar and unipolar resistive switching, respectively [108]. The difference of the switching mode is attributed to different migration or diffusion rates of Ag ions in SnO_2_ and InGaZnO_x_. Compared to single layered SnO_2_ and InGaZnO_x_ devices, both bilayered devices present low operation voltages, high ON/OFF ratios and excellent endurance and retention characteristics. It deserves mention that compared to unipolar operation mode, bipolar mode is preferable for synapse applications given better endurance performance [109]. The reason is explained as follows. If the device is RESET under a voltage bias with the same polarity as that during the SET process (unipolar mode), metal ions will gather again on the tip of the remaining filament resulting in an increase of the concentration of metal ions in the disconnected region. Therefore, the device cannot retain the OFF-state after repeated operations. In contrast, when the device is RESET under a voltage bias with the opposite polarity (bipolar mode), the RESET operation will promote the migration of metal ions away from the remaining filament, thus reducing the metal ion concentration in the disconnected region. In addition, a battery-like architecture of LiCoO_2_/SiO_x_/TiO_2_ has been proposed to mimic biorealistic synaptic behaviour [110]. LiCoO_2_, SiO_x_ and TiO_2_ act as a resistive switching cathode layer, an electrolyte layer and an anode layer, respectively (Figure 3(a)). This device shows analog resistance modulation based on voltage-driven regulation of Li ion concentration in the cathode and anode layers. Synaptic plasticity including short-term potentiation/depression (STP/STD), long-term potentiation/depression (LTP/LTD) and spike-timing-dependent plasticity (STDP) is controlled by the Li ion concentration and its relaxation dynamics (Figure 3(b**,c)**).

### 2.3. Carrier trapping/detrapping

Commonly, memristive synapses based on oxygen or metal ion migration demonstrate relatively large variations of device performance over repeated operation between potentiation and depression due to ion migration-induced microstructure change. Large performance variations are harmful to practical applications of memristive synapses. Theoretically, no microstructure change will occur in the devices based on the resistive switching mechanism of carrier trapping (ON-state) and detrapping (OFF-state) [111,112]. Herein, ‘carrier’ refers in particular to electronic carrier, i.e. electron or hole. Compared to ionic devices, such purely electronic devices are preferable given their potentially better stability.

**Figure 4.**
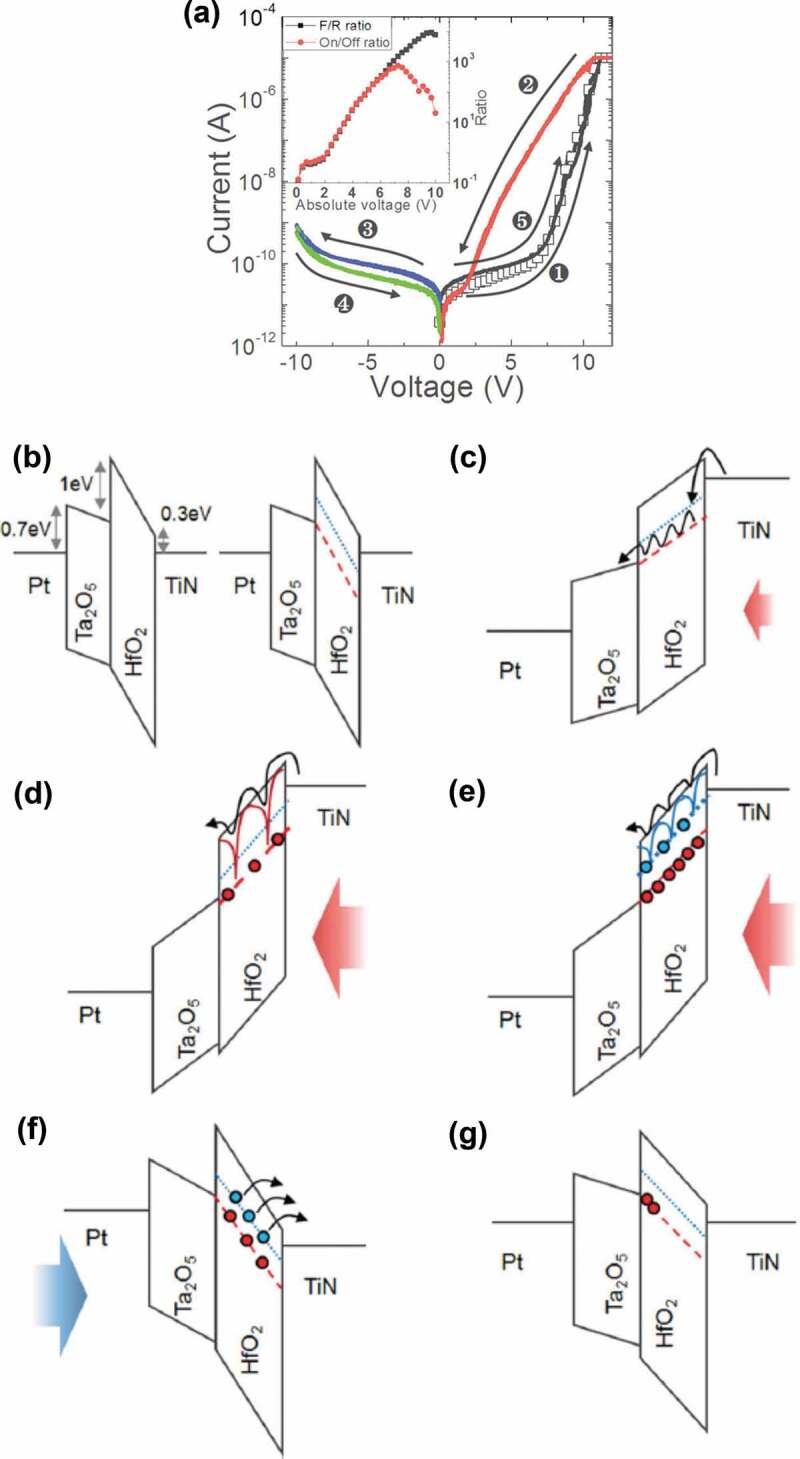
Schematic illustration of the resistive switching mechanism of HfO_2_/Ta_2_O_5_ memristors. (a) Typical I–V characteristics of a device. The inset shows the ON/OFF ratio and rectification ratio as a function of voltage. (b) Band structure for zero bias condition (left panel). The bulk of HfO_2_ has the 1.0 eV traps of oxygen vacancies. The local portion of HfO_2_ has the 0.6 eV traps as well as the 1.0 eV traps (right panel). (c) Under the low positive bias, the injected electrons transport via the hopping mechanism. (d) When the traps with 1.0 eV are filled with electrons, the Poole-Frenkel effect starts to play a role leading to decreased effective trap depth at the higher positive bias. (e) After the deeper trap level is fully filled with electrons, the carrier transport is dominated by the Poole-Frenkel mechanism mediated by 0.6 eV traps. Then the device is switched to an ON-state. (f) When applying negative bias to the device, the electrons in the HfO_2_ traps are detrapped and then the device is switched back to the OFF-state. (g) Under zero bias condition, some of the electrons accumulated at the Ta_2_O_5_/HfO_2_ interface might transport to the empty deep traps and degrade the OFF-state with time. Reproduced with permission [113]. Copyright 2014, Wiley-VCH

A bilayered structure of HfO_2_ and Ta_2_O_5_ can be used to construct purely electronic memristors with a self-rectifying behaviour [113–115]. The HfO_2_ layer serves as the resistive switching layer and the Ta_2_O_5_ layer provides the rectification. The resistive switching mechanism is schematically illustrated in Figure 4. The devices are electroforming-free and show excellent resistive switching uniformity and high rectification ratio. As we know, a memristor crossbar array consists of a set of parallel top electrodes, a set of perpendicular bottom electrodes, and resistive switching material sandwiched between bottom and top electrodes. Each crosspoint represents a memristive device. Sneak paths are an inherent disadvantage of crossbar arrays. As an example [116], the addressed device in the centre of the array is in a relatively high resistance state, whereas all surrounding devices are in relatively low resistance states. When reading, apart from the current flowing through the addressed device, a significant current also flows through the adjacent devices. These two currents cannot be distinguished from one another. It follows that one cannot obtain the correct resistance state of the addressed device. Furthermore, the current and voltage drop across the addressed device is dependent on the current flowing through sneak paths. Therefore, the sneak paths limit the size of a crossbar array [116]. The self-rectifying memristors are able to address the sneak path problem without extra selector devices, thus simplifying the circuits significantly. Besides, purely electronic memristors can be realized using a stack of GdO_x_/Cu-doped MoO_y_ [117]. The resistive switching may be attributed to a carrier trapping/detrapping of the trap sites in the GdO_x_ layer.

## 3. Multilayers of oxides and other materials

### 3.1. Oxygen ion migration

MoO_x_/MoS_2_ and WO_x_/WS_2_ memristive devices have been fabricated by a solution processing method [118], in which MoO_x_ and WO_x_ were obtained by the thermal oxidation of MoS_2_ and WS_2_, respectively. The devices demonstrate highly repeatable bipolar resistive switching with a large ON/OFF ratio up to 10^6^ and multilevel capability. The switching mechanism can be attributed to a Schottky junction modulated by the field-driven migration of oxygen ions at the electrode/oxide interface.

**Figure 5.**
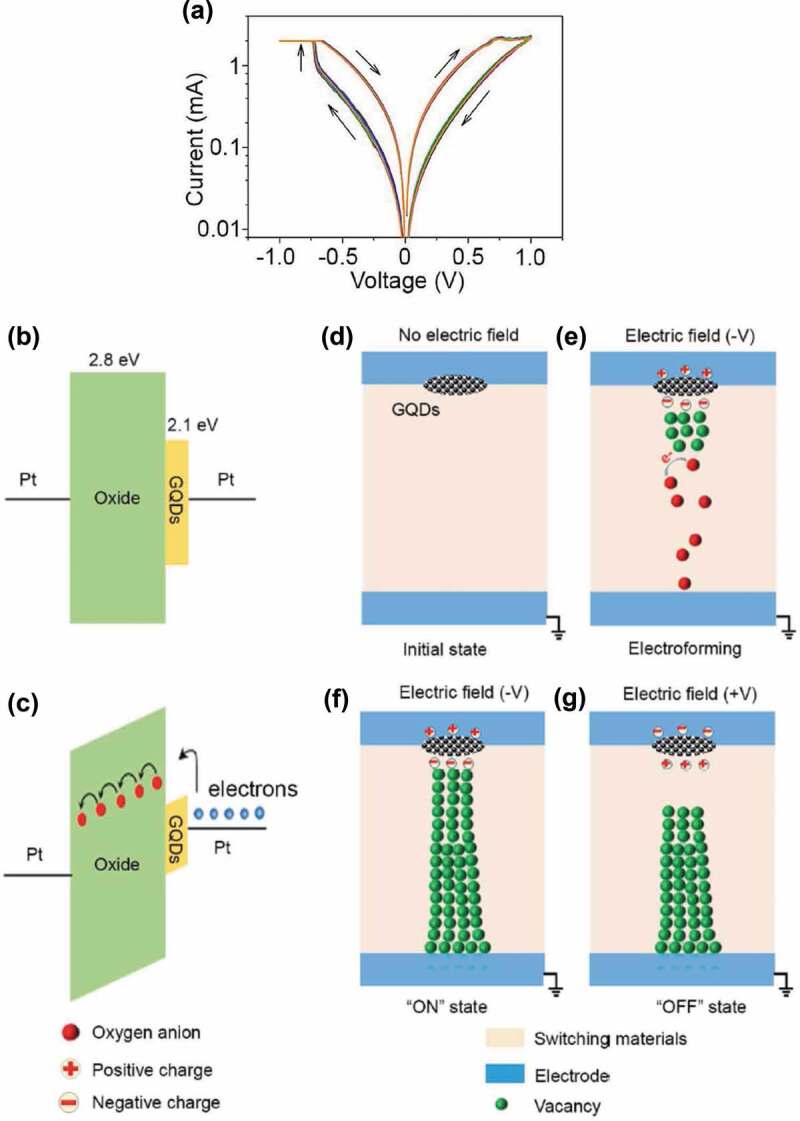
Schematic illustration of the resistive switching mechanism of GQD/FeO_x_ memristors. (a) Typical I–V characteristics of a device. (b) Equilibrium band energy diagram of the device. The bandgaps of FeO_x_ and GQDs are 2.8 and 2.1 eV, respectively. (c) Energy band diagram under a bias. (d) The pristine device. (e) During the electroforming process, oxygen ions (red dots) released from GQDs facilitate the growth of the conducting filament composed of oxygen vacancies (green dots). (f) When the filament connects the bottom and top electrodes, the device is switched to an ON-state. (g) During the RESET process, the filament is ruptured around GQDs switching the device back to the OFF-state. Reproduced with permission [119]. Copyright 2017, Wiley-VCH

Introducing graphene quantum dots (GQDs) into oxide memristors can also effectively improve the device reliability. For example, GQDs in GQD/FeO_x_ memristors serve as nano oxygen-reservoirs and therefore the formation and migration of oxygen vacancies can be manipulated through the oxygen ions released from GQDs [119]. The detailed resistive switching mechanism is schematically illustrated in Figure 5. The device indicates narrowly distributed switching voltages and highly controllable synaptic weight changes.

In addition, a moisture-powered memristor based on a bilayer structure of WO_x_/oxygen-plasma-treated amorphous-carbon (OAC) has been developed [120], in which the WO_x_ and OAC films serve as the resistive switching layer and nanogenerator, respectively. The reversible migration of oxygen ions across the WO_x_/OAC interface allows simultaneous modulation of resistance state and open circuit voltage. Moisture-powered reading of the resistance state can be realized through human breath. The device also demonstrates the capability of the selective reading of multiple resistance states.

### 3.2. Metal ion migration

In a Cu_x_S/WO_y_ memristor [121,122], WO_y_ acts as the resistive switching layer where the formation/rupture of Cu filament occurs whereas Cu_x_S serves as a voltage divider suppressing the large voltage drop in WO_y_. Then an optimum programming energy can be applied to the WO_x_ layer during the depression process, resulting in well-controlled dissolution of Cu filaments. Therefore, linear resistance modulation under identical pulses is realized. Such analog switching behavior during the depression process can be employed for neuromorphic computing. A high pattern classification accuracy is obtained using these Cu_x_S/WO_y_ memristive synapses.

PbS quantum dots (QDs) can be used to improve the uniformity of resistive switching parameters of Ga_2_O_3_ memristors [123]. The enhanced performance is attributed to the ordered arrangement of the PbS QDs which can guide the growth of Ag filaments. The PbS QD/Ga_2_O_3_ device presents a low threshold voltage, uniformly distributed SET/RESET voltages, robust retention, fast response time, and low power consumption. Synaptic functions, e.g. STDP, are mimicked in such PbS QD/Ga_2_O_3_ devices.

Other multilayered structures such as Al_2_O_3_/Ag/ZnO [124], HfO_2_/Ta/Cu_2_S [125], ZnO/Ag nanowire [126], ZrO_2_/WS_2_ [127], TiN/HfAlO_x_ [128] and graphene oxide QD/HfZrO_x_ [129] have also be adopted to control the growth of Ag or Cu filaments. In addition, a bilayer structure of LiCoO_2_/a-Si has been used to control the Li ion motion in battery-like memristive devices [130,131].

### 3.3. Charge trapping/detrapping

An ultrathin memristive synapse based on an Al_2_O_3_/GQD/Al_2_O_3_ structure demonstrates a high performance uniformity and low power consumption [132]. The resistive switching originates from the electron trapping and detrapping of GQDs. Apart from the purely electronic switching mechanism, the high device-to-device and cycle-to-cycle uniformity is also attributed to accurately controlled thickness of the ultrathin Al_2_O_3_ film prepared using atomic layer deposition technique and to the uniformly distributed GQDs on the surface of the Al_2_O_3_ film. Similarly, an Al_2_O_3_/Au/Al_2_O_3_ structure has also been proposed for high performance memristive synapses [133].

It is interesting that the single layered SiN_x_ memristor demonstrates bipolar resistive switching of filamentary mechanism, whereas the bilayered SiN_x_/Al_2_O_3_ device shows a switching mechanism of trapping and detrapping of the electrons in the trap sites of SiN_x_ [134]. The SiN_x_/Al_2_O_3_ memristor exhibits forming-free, self-rectifying and nonlinear characteristics, which are favourable for constructing the crossbar arrays.

## 4. Element-doped oxides

### 4.1. Oxygen ion migration

Mn-doped HfO_x_ [135,136], Al-doped HfO_x_ [137,138], Y-doped ZrO_x_ [139], N-doped TiO_x_ [140], N-doped ZnO [141] and N-doped InGaZnO_x_ [99] have been proposed for memristive devices based on oxygen ion migration. Element doping can improve the reliability and linearity of synaptic weight. Doping of HfO_2_ with metals can suppress the oxide crystallization and thus preserve an amorphous state, leading to improved device performances [137]. Furthermore, metal-doped HfO_2_ exhibits enhanced retention, which may be ascribed to the role of metal dopant in suppressing the outward diffusion of oxygen vacancies [138]. In the case of nitrogen doping, nitrogen atoms can catch the oxygen ions released during the SET process and confine them near the conducting filaments given that N − O bonds are stronger than O − O bonds [140]. During the following RESET process, these captured oxygen ions will be released back and rupture the filaments composed of oxygen vacancies. Then, the formation and rupture of conducting filaments will occur within such confined region, thus resulting in improved switching reliability.

**Figure 6.**
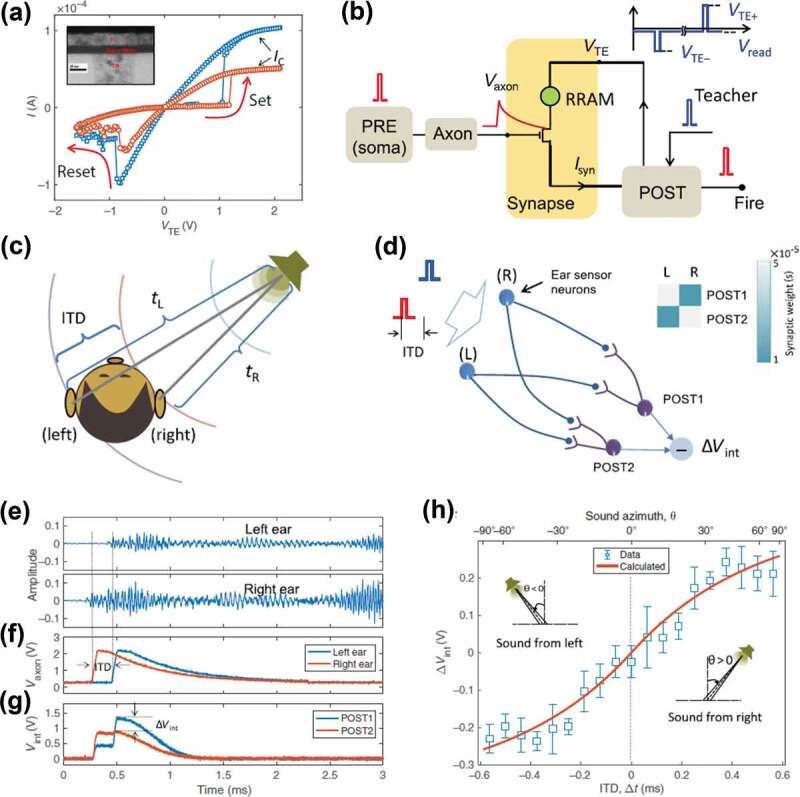
Sound location via a spiking neural network based on Si-doped HfO_x_ memristive synapses. (a) Typical I–V curves of a Si-doped HfO_x_ memristor. The transition to the ON-state is controlled by the compliance current *I*_C_. (b) Schematic illustration of a 1T1M synapse which connects a PRE axon to the POST. The POST provides a feedback spike for potentiation/depression. (c) Schematic illustration of binaural effect. The ITD provides a clue of the direction of the sound propagation. (d) Schematic structure of a 2 × 2 SNN. The difference of *V*_int_ between the two POSTs acts as the output of the network, which provides information about sound direction. The inset presents the map of synaptic weights of the synapse array, enabling discrimination between different directions. (e) Experimental audio signals for left and right ears. (f) The corresponding axon potential of the two PREs. (g) *V*_int_ for the two POSTs. (h) Measured and calculated ∆*V*_int_ as a function of sound direction angle uncovering analog information about the sound propagation direction. Reproduced under the terms of the CC-BY Creative Commons Attribution 4.0 International License [142]. Copyright 2018, The Authors, published by American Association for the Advancement of Science

Memristive synapses based on Si-doped HfO_x_ have been used to construct spiking neural networks (SNNs), combined with suitable neuron circuits and operation schemes [142,143]. The basic properties of a Si-doped HfO_x_ memristive synapse are illustrated in Figure 6(a). To enable time-dependent modulation of the synaptic weight, the memristor is connected with a field-effect transistor in the synaptic circuit (Figure 6(b)). The one-transistor/one-memristor (1T1M) synapse is controlled by the presynaptic neuron (PRE) through the transistor gate terminal and transmits the synaptic current *I*_syn_ to the postsynaptic neuron (POST) through the transistor source terminal. The axon terminal transforms the PRE spike into an exponentially decaying pulse *V*_axon_, which controls the transistor gate and then the current. The time delay between the PRE spike *V*_axon_ and the POST spike *V*_TE_ applied to the top electrode of the memristor leads to a time-dependent SET process (synaptic potentiation) of the memristor. It forms the basis of the spatiotemporal processing by the memristive synapse. The SNNs are capable of learning and recognizing spatiotemporal patterns via STDP, such as spike sequences and spike groups in which the spike timing carries information. The spike timing difference among different neurons provides spatiotemporal coding with high information capacity. This work is an important step toward biorealistic machines possessing the energy efficiency and computing functionality of the human brain. As an example, such SNN can be employed to mimic the detection of the sound location by the brain. As schematically illustrated in Figure 6(c), the human brain detects sound location by the interaural time difference (ITD), i.e. *t*_L_ – *t*_R_, where *t*_L_ and *t*_R_ are the times of the sound reaching the left and right ears, respectively. The SNN comprises a fully connected network of two PREs, corresponding to sensors in each ear, and two POSTs, with 2 × 2 memristive synapses (Figure 6(d)). The synaptic weight matrix is set to be diagonal, that is, one POST fires responding to the left or right pattern, whereas the other fires responding to the opposite sequence. Then the difference of the internal potentials (*V*_int_) between two POSTs provides an indication of the sound direction angle. Figure 6(e–g) shows an example of detecting the sound location. Audio signals for the left and right ears (Figure 6(e)) induce the spikes of the corresponding PREs (Figure 6(f)). The PREs respond to the first detected wave front and send the spatiotemporal spiking pattern to the network. The difference of *V*_int_ between POST1 and POST2 at the time of the second PRE spike indicates the sound direction angle (Figure 6(g)). The positive/negative ∆*V*_int_ indicates that the sound reaches the right/left ear first, and therefore the origin of the sound is from the right/left side of the receiver. Figure 6(h) illustrates the relationship between ∆*V*_int_ and ITD. The results demonstrate that the SNN has the capability of converting external analog stimuli into inner-network representation through spatiotemporal coding.

### 4.2. Metal ion migration

Cu-doped SiO_2_ has been used to fabricate memristive synapses with a working mechanism of Cu ion migration [144]. The device resistance can be gradually modulated in both directions by voltage sweeps or pulses. The gradual change of resistance is attributed to the expansion or contraction of a Cu-rich oxide layer. It is found that the magnitude of voltage pulses play a more important role in resistance change than pulse width, especially in the high voltage region. This result provides important guidance for circuit design. TiO_2_ films with self-assembled Ag nanoclusters implemented by gradient Ag dopant have also been adopted to obtain enhanced memristor performance [145]. Besides, Ag-doped Ta_2_O_5_ [146], Ag-doped HfO_x_ [147], Ag-doped MgO_x_ [147], Ag-doped SiO_x_N_y_ [147], Cu-Al-codoped SiO_2_ and Cu-Ga-codoped SiO_2_ [148] have been employed to fabricate memristive devices based on metal ion migration.

Cu or Ag doping into oxide layers can effectively reduce the electroforming voltage and improve the following resistive switching [144]. Al and Ga doping can increase the solubility of the Cu ions in SiO_2_. It is because that Al^3+^ and Ga^3+^ ions form negatively charged cation point defects by substituting Si^4+^ and serve as a counter charge of Cu^x*+*^, thus allowing excessive Cu ions to remain dissolved/unclustered in SiO_2_ [148].

## 5. Composite oxides

**Figure 7.**
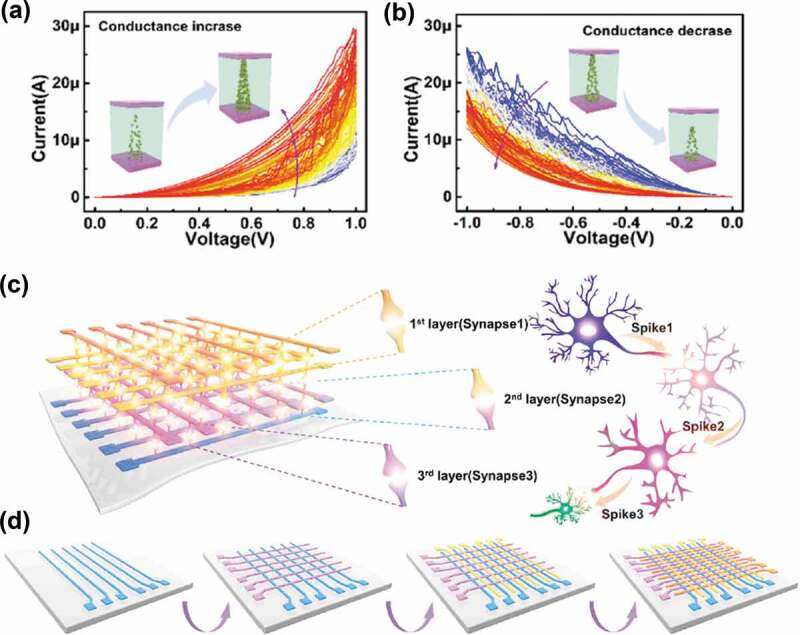
Schematic illustration of a 3D artificial neural system based on HfAlO_x_ memristors. (a) (b) Typical I − V curves of a device under consecutive positive and negative biases. The inset schematically illustrates the growth and rupture of oxygen vacancy filament in the device. (c) Structure of an artificial neural network comprising trilayer crossbar memristor arrays. (d) The fabrication process of the 3D network on flexible polyethylene terephthalate. Reproduced with permission [149]. Copyright 2020, American Chemical Society

A flexible three-dimensional (3D) artificial neural network made of three-layer HfAlO_x_ memristor crossbar array on polyethylene terephthalate has been developed (Figure 7) [149]. The memristor works based on the migration of oxygen ions and presents ultralow energy consumption of ~4 aJ/spike, even far lower than that of the biological system with an energy consumption of 1–10 fJ/spike. Based on such synaptic network, the multilevel information transmission between synapses of different layers is simulated. Information can be transferred through the selected synapses. Synaptic weight modulation including LTP and LTD can be realized in such 3D arrays under a flat and bending state, thus indicating excellent flexibility of the network.

Furthermore, a high performance HfAlO_x_ memristive device with TiN as the electrode has been developed by introducing an electro-thermal modulation layer [70]. The device presents excellent cell-to-cell uniformity and high multilevel reliability. Such device can be used to implement a Hopfield neural network (HNN). As we know, associative memory is an important ability for humans, for example, recalling memories from partial information. Different models have been proposed to mimic the associative memory function via an artificial intelligence system, e.g. HNN. The HNN is a fully connected auto-associative network where stable states are the local minimum of the energy function. In HNN, a target pattern can be memorized as a stable state. As incorrect or incomplete pattern is inputted into the network, the original pattern can be recalled. Hence, HNN can be employed for image processing and prediction. The HNN made of HfAlO_x_ memristors demonstrates tolerance to load error and read noise and is capable of emulating the associative memory for emotion image recovery [70]. Given that HNN is a kind of artificial neural networks and memristors serve as artificial synapses in HNN, memristive devices demonstrating non-volatile multi-level resistive switching behaviour are needed [70,150].

HfZnO_x_ [151] and HfZrO_x_ [152] composites have also been proposed for memristive synapse application with inert metals (Pt and Au) as the electrode.

In a HfZrO_x_ memristor using active Cu electrode [153], the quantum conductance phenomenon has been observed which can be ascribed to interstitial Cu in the HfZrO_x_ layer forming single- and multi-atom chains. The gradual resistance tuning enables the device to mimic synaptic functions such as memory and learning. In a Ag/HfZrO_x_: graphene oxide QD/Ag memristor [129], the pulses with low energy can realize almost linear resistance modulation, which is favourable for increasing the accuracy of neuromorphic computing. The gradual resistance tuning might be ascribed to the coexistence of Ag ion migration and tunneling effect.

A bilayered Ag/TiN/HfAlO_x_/Pt memristor exhibits a large selectivity (10^10^), a wide current range from 10 nA to 1 mA and an extremely steep slope (0.63 mV/dec) [128]. The stable threshold switching might be attributed to the alternate atomic layer deposited HfAlO_x_ layer and the introduction of TiN buffer layer. Combined with a RC circuit, the device can mimic the leaky integrate-and-fire function of neurons with low power consumption. The leaky integrate-and-fire function can be concisely described as follows. A neuron is in a resting state without external stimuli, i.e. not conducting any impulse. If the external stimuli are not strong enough, the neuron maintains the resting state. When the stimuli accumulate and exceed a threshold value, action potentials are generated. After that, the neuron is restored to its initial resting state. Therefore, compared to memristive devices showing non-volatile memory switching behaviour, the devices with volatile threshold switching are preferable for neuron applications [14,128,154].

## 6. Other hybrid oxides

**Figure 8.**
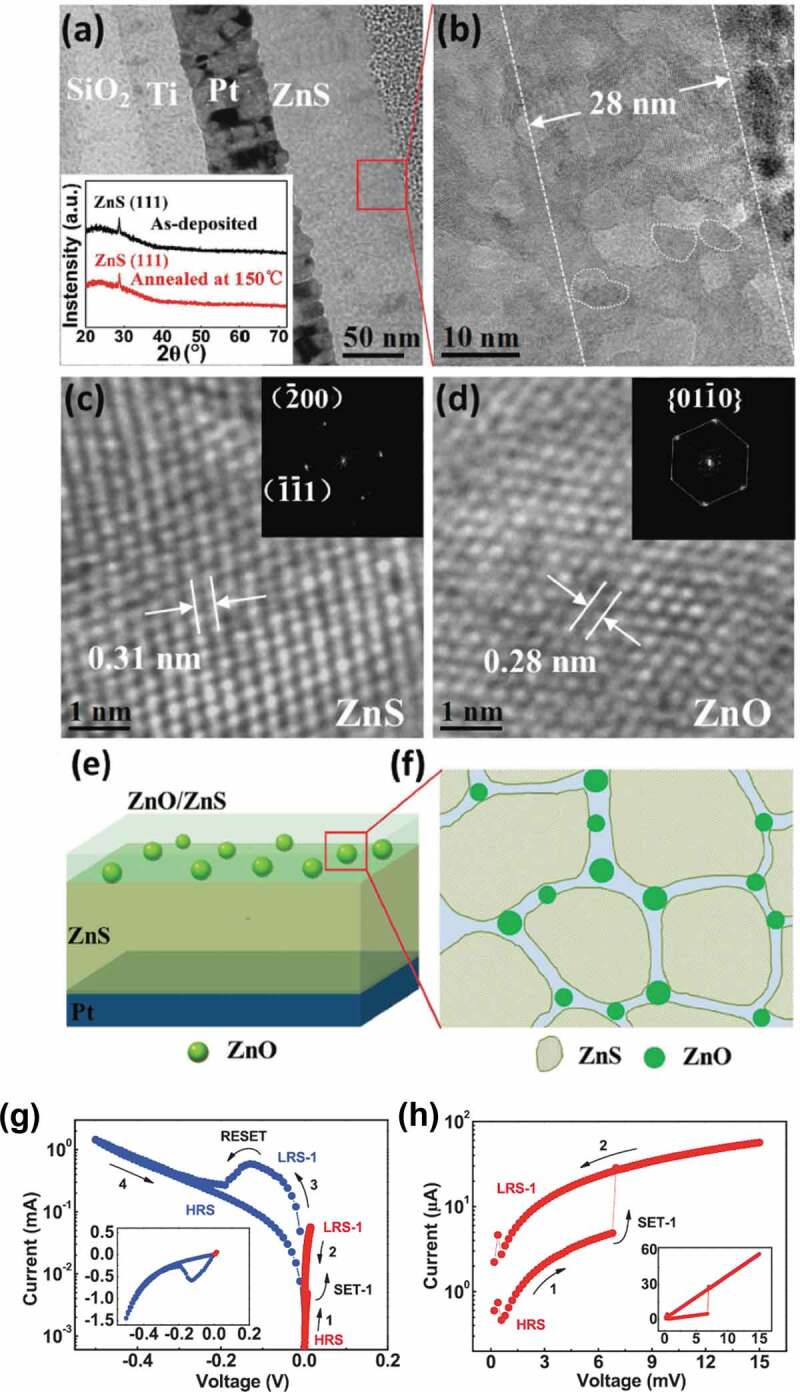
Microstructure and resistive switching behavior of lightly oxidized ZnS films. (a) Cross-section transmission electron microscopy image of the films. The inset presents the X-ray diffraction patterns of as-deposited and oxidized films. (b) The high-resolution transmission electron microscopy image for the area marked by the red rectangle in (a). (c,d) Lattice images for most of the image area and the areas marked by white dotted lines in (b). The insets show the corresponding fast Fourier transform images. (e) Schematic illustration of the bilayered structure of oxidized films. (f) Schematic enlargement of the area marked by the red rectangle in (e). (g) Typical I − V curves of a device. The inset presents the curves on a linear scale. (h) The enlarged I − V curve for the SET process. The inset presents the curve on a linear scale. Reproduced with permission [155]. Copyright 2017, Wiley-VCH

Memristive devices based on lightly oxidized ZnS films (Cu/lightly oxidized ZnS/Pt) demonstrate highly controllable resistive switching with an ultralow SET voltage of several millivolts [155]. The distinctive device performance can be ascribed to a bilayer structure of the lightly oxidized and unoxidized ZnS (Figure 8). Light oxidation results in a small number of ZnO nanocrystals dispersed in the ZnS matrix. The Cu filament rupture/rejuvenation will be confined to the bilayer interface due to different Cu ion migration rates in these two layers. An ultrasensitive memristive synapse is then achieved where the synaptic functions such as STP and LTP are mimicked by applying electrical pulses several millivolts in amplitude. The sensitivity of such devices surpasses that of biological synapses. It should be noted that the structure of such lightly oxidized ZnS films is different from that of thermally oxidized few-layer MoS_2_ or WS_2_ flakes (see Ref [118].). The former contains ZnO nanocrystals surrounded by the ZnS matrix, whereas the latter is composed of MoO_x_/MoS_2_ or WO_x_/WS_2_ bilayer.

Nitride oxides can also be introduced into memristors, acting as a function or buffer layer. The oxidation of atomically thin two-dimensional BN leads to an amorphous BNO_x_ material [156,157]. The resulting device demonstrates a filamentary resistive switching behavior with sub-pA operation current and fJ/bit energy consumption. By confining the Ag filament to the atomic scale, current switching characteristics can be observed, much different from that in thicker switching layer likely due to the significantly different atomic kinetics. By inserting a TiNO_x_ buffer layer [158–160], oxide memristors exhibit effectively improved reliability and reduced power consumption. AlNO_x_ [161] and SiO_x_N_y_ [147] have also been used as the function layer in filamentary oxide memristive devices.

In addition, AgI/Al_2_O_3_ composite electrolytes are proposed for memristors [162]. Compared to pure AgI devices, the composite devices can form initial Ag filaments significantly faster with a relatively low ON/OFF ratio.

## 7. Discussion and conclusions

Due to a rather broad application space of memristive synapses, it is difficult to define generalized metrics for memristive synapses. By referring to the properties of biological synapses, some desired characteristics for memristive synapses can be summarized as follows [163]. A device size <20 × 20 nm^2^ and an energy consumption per synaptic event <10 fJ are needed for the construction of artificial neural networks with an ultrahigh-density integration of synapses comparable to the human brain. Given the heat dissipation problem, this ultralow energy consumption is crucial for developing ultrahigh-density neuromorphic circuits. Although many memristive synapses show operation frequencies >10 MHz, an operation frequency of 10 Hz is preferable considering the tradeoff between energy consumption and operating speed. A programming time <1 ms is enough to realize synaptic plasticity. Due to the variety in biology, it is difficult to specify the number of multi-level resistance states required for memristive synapses by referring to biological ones. Anyway, more multi-level states are preferable given the capacity and robustness of neural networks. 20‒100 resistance states are recommended for general synapse applications. And a dynamic resistance range >4 is required where the dynamic range is defined as the maximum resistance ratio of the highest-resistance state to the lowest-resistance state. In biological synapses, long-term plasticity lasts from hours to years. A retention of 10 years is preferable for each resistance state of memristive synapses after the operation of LTP or LTD to enable formation of long-term memories. An endurance of 3 × 10^9^ synaptic operations can ensure 10 years of lifetime with an operation frequency of 10 Hz. Neuromorphic networks, to a certain extent, promise immunity against device variations [163–166]. The variation degree, which can be tolerated in the network level, is strongly dependent on the network structure and the required application accuracy [163]. The simulation results show that for pattern completion via associative recall, although the success rate decreases with increased variation of device resistance, a success rate >80% can be obtained even the device shows a resistance variation as large as 30% [164]. As for a neuromorphic visual system, no degradation is observed in the system performance when the experimental device variability level of 9% is introduced to simulations, and further raising the variability causes a slight degradation [165].

Oxide memristive synapses are the most promising candidate for brain-inspired neuromorphic computing. Generally, the resistive switching mechanisms of oxide memristors are based on metal or oxygen ion migration, that is, stochastic formation/rupture of nanoscale conducting filaments. Such working mechanisms result in large device-to-device and cycle-to-cycle performance variations, thus becoming a key obstacle to realize large scale artificial neural networks. Hybrid oxides, including multilayers of oxides, multilayers of oxides and other materials, element-doped oxides and composite oxides, can effectively enhance the device stability and reliability given highly controllable ion migration and confined formation/rupture of conducting filaments. Compared to filamentary devices, purely electronic memristors based on carrier trapping/detrapping can possess better stability and reliability considering that no microstructure change occurs during the operation [113]. Furthermore, purely electronic devices always exhibit a self-rectifying behaviour, which can be utilized to address the sneak path problem when constructing memristor crossbar arrays. Hence, it could be argued that oxide memristors with a resistive switching mechanism of carrier trapping/detrapping are preferable for synapse applications given their relatively high working stability and reliability. Filamentary devices showing relatively stochastic operating parameters could be used as artificial neurons since stochastic neuronal dynamics are crucial in signal encoding and transmission [167].

Although significant progress has been made in the development of memristive synapses, the relevant research field is still in its incipient stage. The device stability and reliability are far from meeting the standard of practical applications in neuromorphic computing. It remains a big challenge to construct a highly effective artificial neural network with relatively low density, e.g. k bits, based on oxide memristors. In most cases, memristors are used to mimic deep neural networks. Spiking neural networks mainly based on STDP, that reflect the actual information processing style in the human brain, are seldom investigated [142]. Anyway, oxide-based hybrid structures provide a promising prospect for the application of oxide memristors to neuromorphic computing and therefore hardware implementation of artificial intelligence.
